# 391. Virologic Outcomes with Lenacapavir in Heavily Treatment-Experienced People with HIV: A Multi-Center Real-World Study

**DOI:** 10.1093/ofid/ofaf695.129

**Published:** 2026-01-11

**Authors:** Christopher Kaperak, Yijia Li, Maria F Sanes Guevara, Beverly E Sha, Shivanjali Shankaran, Mariam Aziz, Nancy Glick, Bijou R Hunt, Cathy Creticos, Paul S Djuricich, Andrew Merker, Aniruddha Hazra

**Affiliations:** University of Chicago Medicine, Chicago, IL; University of Pittsburgh, Pittsburgh, Pennsylvania; University of Pittsburgh, Pittsburgh, Pennsylvania; Division of Infectious Diseases, Department of Internal Medicine, Rush University Medical Center, Chicago, IL, USA, Chicago, Illinois; Rush University Medical Center, Chicago, Illinois; Rush University Medical Center, Chicago, Illinois; Sinai Infectious Disease Center, Chicago, Illinois; Sinai Infectious Disease Center, Chicago, Illinois; Howard Brown Health, Chicago, Illinois; University of Chicago Medicine, Chicago, IL; UChicago Medicine, Chicago, Illinois; University of Chicago, Chicago, IL

## Abstract

**Background:**

Lenacapavir (LEN) is a novel long-acting injectable antiretroviral therapy (ART) drug for heavily treatment-experienced (HTE) people with HIV (PWH). However, real-world data on its use and outcomes are limited. We describe early real-world experience with LEN among HTE PWH across five clinics in Chicago, IL and Pittsburgh, PA.

**Figure:**
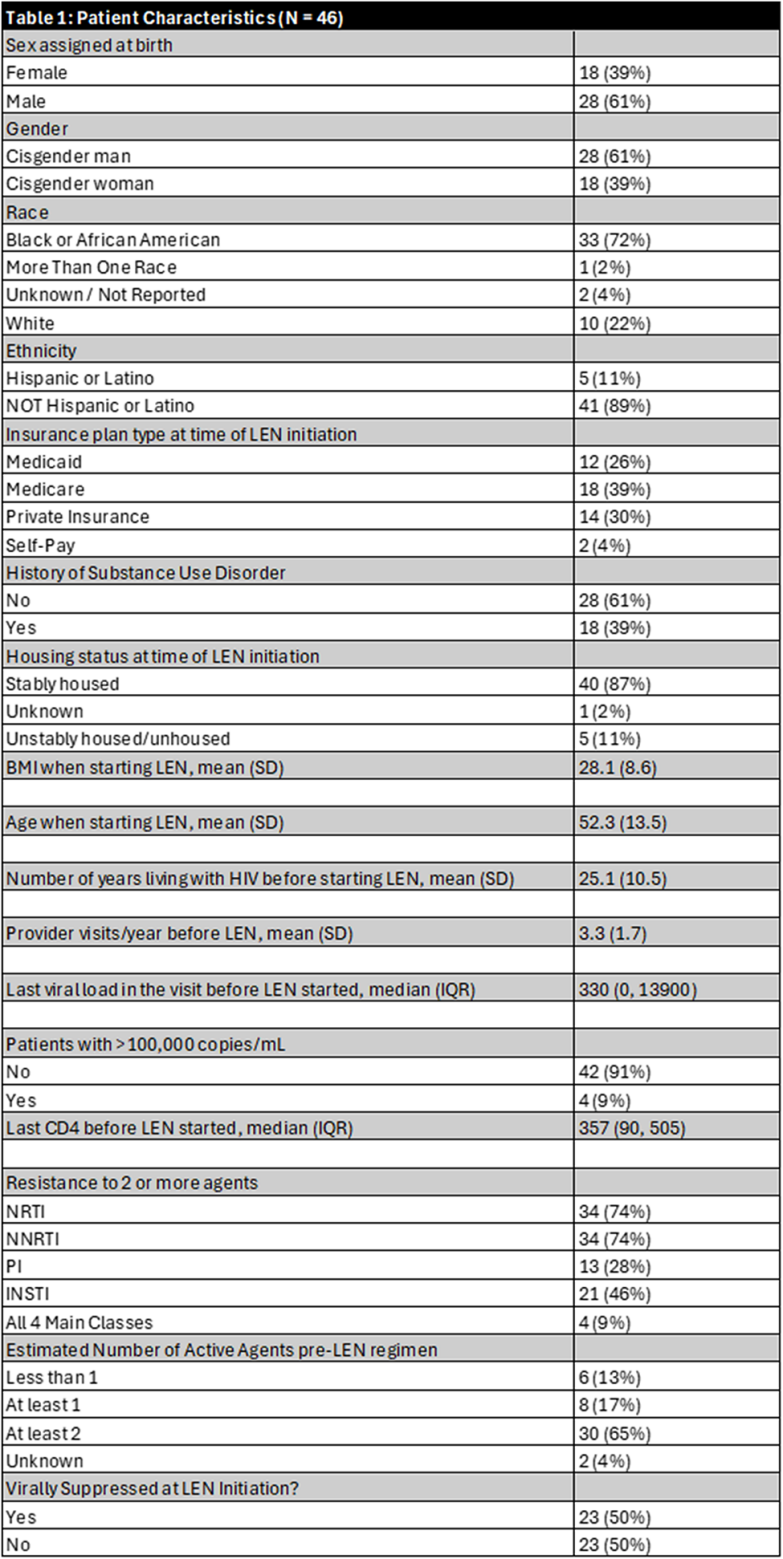

**Figure:**
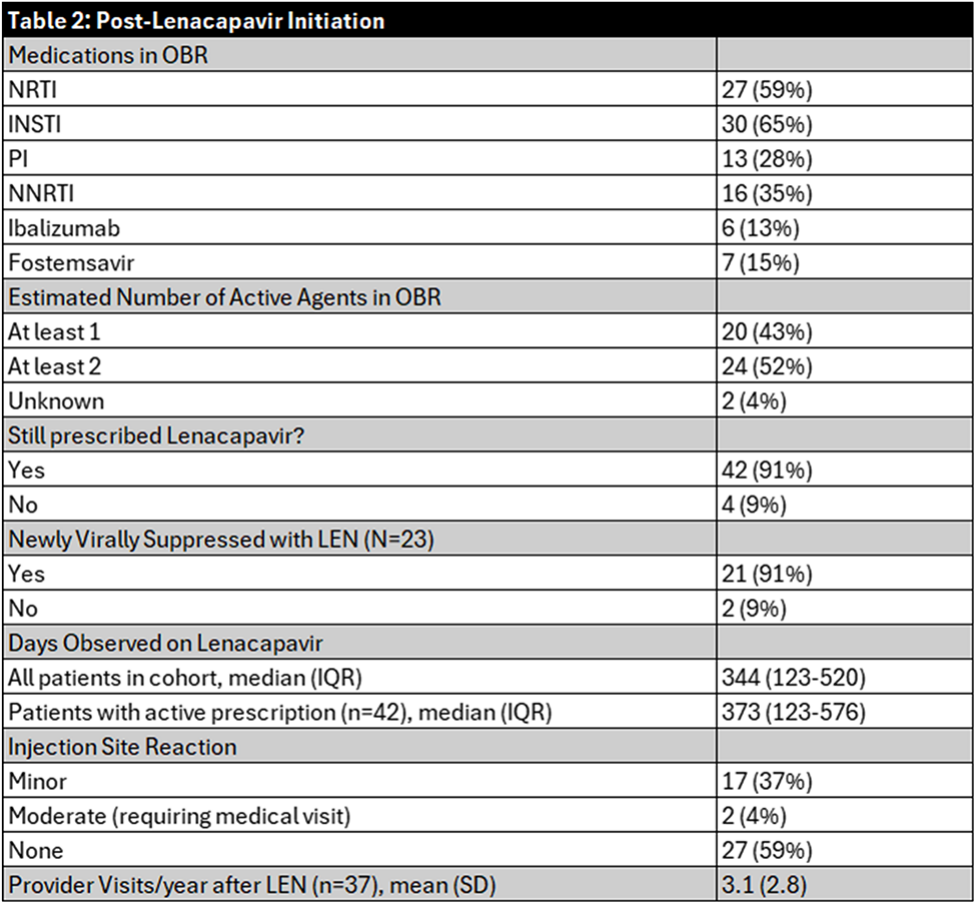

**Methods:**

We conducted a multi-center retrospective cohort study of HTE PWH who started LEN from November 13, 2020, through April 4, 2025. Demographic and clinical characteristics were collected, including previous ART regimens, HIV drug resistance mutations, HIV viral loads (VL), and self-reported adherence to optimized background regimen (OBR) after starting LEN.

**Figure:**
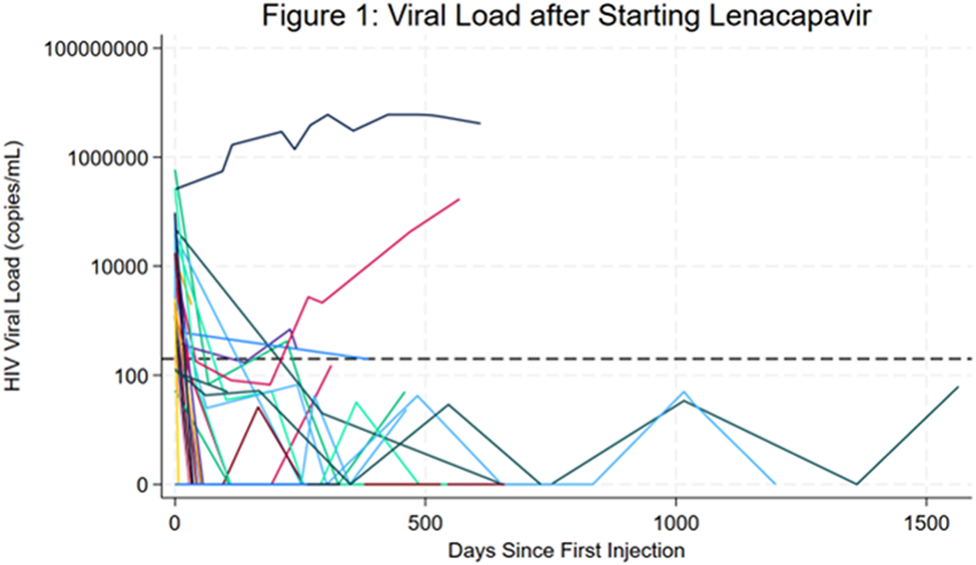

**Figure:**
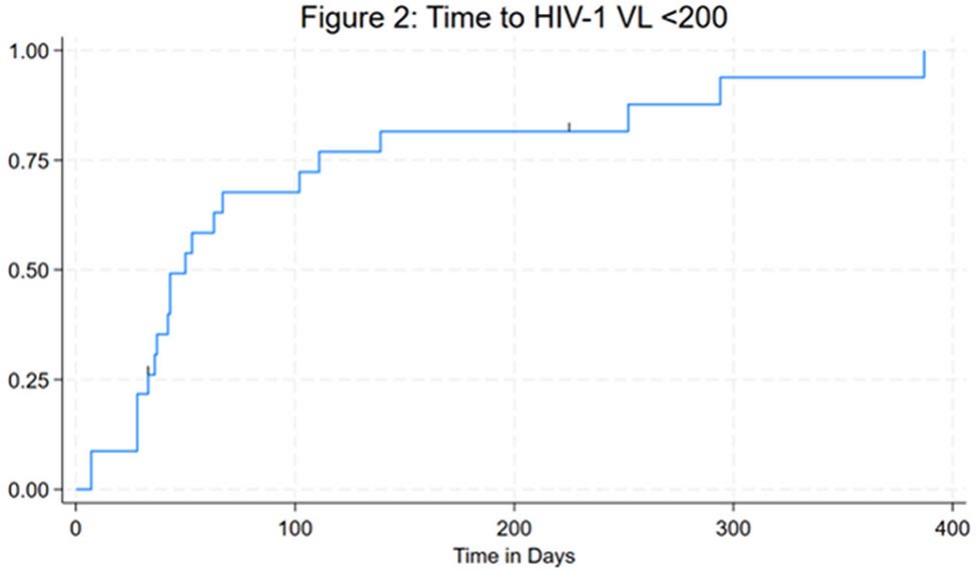

**Results:**

Forty-six individuals received at least one dose of LEN; most were cisgender men (61%), Black (72%), and had stable housing (87%) (Table 1). At initiation, their mean age was 52 years and on average had been living with HIV for 25 years. Extensive drug resistance was common: 34 had resistance to ≥2 NRTIs, 34 to ≥2 NNRTIs, 13 to ≥2 PIs, and 21 to ≥2 INSTIs; four showed resistance across all four drug classes. Among 23 individuals with viremia at LEN initiation (VL ≥200 c/mL), 91% achieved viral suppression during the observed period, with 70% doing so within the first 3 months (Figures 1 and 2). Two individuals remained unsuppressed after LEN due to self-reported OBR non-adherence. All individuals without viremia at initiation maintained viral suppression throughout follow-up. Before LEN, 30% had a genotypic susceptibility score indicating their regimen had fewer than two fully active agents; after initiation, all had regimens with at least two active agents (Table 2). Nearly all individuals (91%) remained on LEN through the observation period; median time on LEN was 373 days (IQR 123–576). Discontinuations (n=4) were due to OBR non-adherence, injection site reaction, hospice entry, or loss to follow-up.

**Conclusion:**

In this real-world cohort, LEN-based regimens resulted in high rates of virologic suppression among HTE PWH. Our findings support LEN as a promising salvage therapy when paired with an active OBR. As LEN use expands, targeted implementation strategies will be critical to address adherence challenges, manage side effects, and optimize sustained treatment success for HTE populations.

**Disclosures:**

Aniruddha Hazra, MD, Abbott Laboratories: Advisor/Consultant|Gilead Sciences, Inc: Advisor/Consultant|Gilead Sciences, Inc: Grant/Research Support|GSK/ViiV Healthcare: Advisor/Consultant

